# Sex-related differences on the risks of in-hospital and late outcomes after acute aortic dissection: A nationwide population-based cohort study

**DOI:** 10.1371/journal.pone.0263717

**Published:** 2022-02-10

**Authors:** Fang-Ting Chen, An-Hsun Chou, Yi‐Hsin Chan, Victor Chien-Chia Wu, Chia-Pin Lin, Kuo-Chun Hung, Pao-Hsien Chu, Yu-Ting Cheng, Shao-Wei Chen

**Affiliations:** 1 Department of Anesthesiology, Linkou Medical Center, Chang Gung Memorial Hospital, Chang Gung University, Taoyuan City, Taiwan; 2 Department of Medicine, Chang Gung University, Linkou, Taipei, Taiwan, ROC; 3 Department of Anesthesiology, Xiamen Chang Gung Hospital, Taoyuan, Taiwan; 4 Department of Cardiology, Linkou Medical Center, Chang Gung Memorial Hospital, Chang Gung University, Taoyuan City, Taiwan; 5 Division of Thoracic and Cardiovascular Surgery, Department of Surgery, Linkou Medical Center, Chang Gung Memorial Hospital, Chang Gung University, Taoyuan City, Taiwan; 6 Center for Big Data Analytics and Statistics, Linkou Medical Center, Chang Gung Memorial Hospital, Taoyuan City, Taiwan; Imperial College London, UNITED KINGDOM

## Abstract

**Objective:**

The aim of this study is to evaluate the sex-related differences on the risks of perioperative and late outcomes for adult acute aortic dissection (AAD) patients following surgical management.

**Methods and results:**

By using Taiwan National Health Insurance Research Database, totally 1,410 female and 3,432 male patients were identified to first-ever receive type A AAD open surgery or type B AAD stenting treatment from 2004 to 2013. We assessed the sex-related difference on outcomes, including in-hospital mortality, all-cause mortality, aortic death, redo aortic surgery, ischemic stroke, and depression during the follow-up period. The analysis was done separately for type A and type B surgeries.

**Results:**

On average, female patients diagnosed with AAD were older than males. There was no significant sex difference of in-hospital mortality or all-cause mortality for both type A open and type B stent surgeries. The risk of redo aortic surgery was significantly greater in males than females (7.8% vs. 4%; unadjusted subdistribution hazard ratio [SHR] 0.51, 95% CI 0.38–0.69) for type A open surgery, but not for type B stent surgery. Noticeably, the risk of newly-diagnosed depression was significantly greater in females than males (8% vs. 5.1%; unadjusted SHR 1.6, 95% CI 1.24–2.06) for type A open surgery, but not for type B stent surgery.

**Conclusions:**

No significant sex-related difference was found for the in-hospital mortality or accumulative all-cause mortality. However, there were more redo aortic surgeries for males and more postoperative depression for females in type A AAD population.

## Introduction

Acute aortic dissection (AAD) is a life-threatening disease with high morbidity and mortality rates, requiring timely treatment. Based on an observational analysis from the International Registry of Acute Aortic Dissections (IRAD), approximately two-thirds (67%) of patients were diagnosed with type A, and the remaining as type B AAD. The patients in both types of AAD were predominantly men; nevertheless, the outcome of female patients was worse [1]. Few studies have indicated differences in demographics, clinical manifestation, diagnostic vascular imaging, managements, and outcomes between the sexes [2–4]. However, most of these studies comprised mostly of small cohorts with varied results. Moreover, some studies has stated sex-related difference on the development of depression, which was further associated with increased risk of heart disease [5, 6]. The difference of incidence for depression between the sexes had also been reported in patients with post-acute coronary syndrome [7].

This population-based cohort study aimed to assess the effect of sex-related differences on the demographic characteristics, perioperative morbidities/mortality rate, and late outcome after aortic dissection.

## Materials and methods

### Data source

This retrospective cohort study utilized data from the National Health Insurance (NHI) Research Database (NHIRD), deprived from the government-operated single-payer NHI program and covers almost all the residents of Taiwan (>99%). The medical costs of all lifesaving treatments, including cardiac surgeries for type A/B AAD, endovascular stent therapy for type B AAD, and perioperative blood transfusion, are reimbursed by the NHI. Numerous studies using data from the NHIRD for analysis have been published [8–11]. In the NHIRD, disease diagnoses are coded based on the International Classification of Diseases, 9^th^ Revision, Clinical Modification (ICD-9-CD). The Ethics Institutional Review Board of Chang Gung Memorial Hospital approved this study.

### Study population

**Fig 1:**
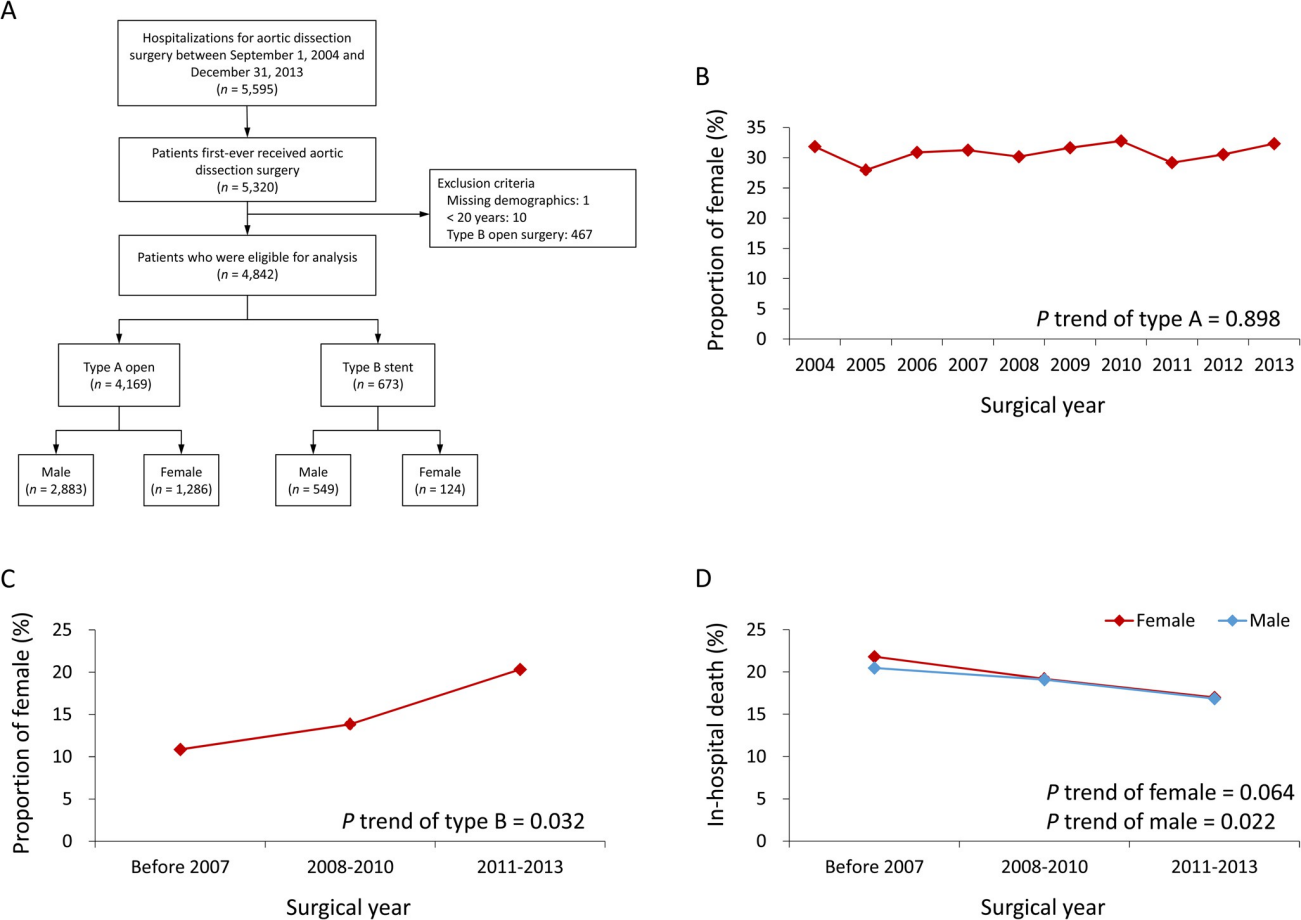
Flow chart for patient selection (A), epidemiology for the trend of sex distribution in receiving type A open surgery (B) and type B stent surgery (C) and the trend of in-hospital mortality among the acute aortic dissection surgeries in the males and females (D) over the study period. Due to the small sample size for type B stent surgery, the surgical year was divided into three points.

In this study, AAD was divided into type A and B according to the Stanford classification regarding the involved site of aortic dissection [12]. We searched the NHIRD using the ICD-9 primary diagnosis code (441.0), combined with other procedure codes for aortic surgeries, to recognize patients with aortic dissection between September 1, 2004, and December 31, 2013 [13]. We excluded patients previously diagnosed with aortic dissection, those with incomplete demographics, and those younger than 20 years. We also excluded patients who underwent open surgery for type B AAD because stent surgery is currently the mainstream therapy for type B AAD [14, 15]. The remaining patients were further subdivided by sex (**Fig 1A**).

### Covariates

The covariates in this study included age, monthly income, urbanization level, history of previous cardiac surgery, 13 comorbidities, Charlson’s Comorbidity Index score, hospital level of the index AAD admission, cumulative hospital volume of aortic dissection surgery (2004─2013), 9 kinds of anti-hypertensive agents, 5 other classifications of medications, surgical details of the AAD surgery, and any additional cardiac surgeries. The comorbidities were identified using at least two outpatient diagnoses or any of the inpatient diagnoses in the year prior to the index AAD admission. Previous cardiac surgery, surgical details of the AAD surgery, and additional cardiac surgeries were detected using the Taiwan NHI reimbursement codes from the inpatient claims data. The medication information was extracted from the prescription records of the claims data from both outpatient visits and pharmacy refills for chronic illnesses during the first 90 days after discharge.

### Outcomes

In-hospital outcomes were in-hospital mortality, new-onset stroke, and massive blood transfusion. Late outcomes included all-cause mortality, redo aortic surgery, and newly diagnosed depression. Death was defined as a withdrawal from the NHI program [16]. Stroke events were detected using the principal diagnosis of the hospitalization. Redo aortic surgery and the amount of blood transfusion were identified using the Taiwan NHI reimbursement codes from the inpatient claims data. Massive blood transfusion was defined as packed red blood cells >10 units. These outcomes have been reported in our previous study [16]. Newly diagnosed depression was verified by both the outpatient diagnosis and any prescriptions of anti-depressants. All patients were followed from the index AAD admission to December 31, 2013, or death, depending on which came first.

The primary analysis of comparing the sex difference on the risks of outcomes used the raw data for real-world practice without propensity score matching (PSM). The analysis using the PSM cohort was the secondary analysis in this study.

### Statistical analysis

The PSM with 1:1 ratio was conducted separately for the type A AAD open surgery and type B AAD stent surgery. The sex difference of the baseline characteristics was evaluated using standardized difference (STD), where an absolute value of STD less than 0.2 is considered small difference. In addition, formal statistical tests (independent sample t-test and chi-square test) were also performed to assess the sex difference in the baseline characteristics. The sex difference of in-hospital outcomes was comparing using univariate linear and logistic regression analyses for continuous and binary outcomes respectively. The sex difference in the risks of time to event outcomes was compared using univariate Cox proportional hazard model for fatal outcomes (i.e., all-cause mortality) or Fine and Gray subdistribution hazard model for non-fatal outcomes (e.g., redo aortic surgery and depression) which considered all-cause mortality a competing risk. A two-sided *P* value <0.05 was considered statistically significant. Statistical analyses were performed using SAS version 9.4 (SAS Institute, Cary, NC).

## Results

### Epidemiology of AAD surgeries in Taiwan

This study included 4,842 patients categorized as either type A AAD with open surgery (N = 4169; male, N = 2883; female, N = 1286) and type B with endovascular stenting treatment (N = 673; male, N = 549; female = 124) from September 1, 2004 to December 31, 2013 (**Fig 1A**).

Approximately 30% of the population in the type A AAD open surgery group were women, and that proportion remained stable over the study period (*P* for trend = 0.898; **Fig 1B**). The proportion of women increased remarkably over time among the population with type B AAD stent (*P* for trend = 0.032; **Fig 1C**).

The in-hospital mortality of the combined AAD surgeries gradually decreased across the study period in both sexes (*P* for trend for females and males = 0.064 and 0.022 respectively; **Fig 1D**).

### Patient characteristics

Women tended to be older than men at the first AAD diagnosis in both groups. The surgical volume among the type B stenting group substantially increased in 2011─2013 (**Table 1**). The leading three comorbidities were hypertension, diabetes mellitus, and chronic kidney disease. The prevalence of depression in female patients was higher than that in male patients (type A AAD, female: 15.9%; type B AAD, female: 16.1%) (**Table 2**). No significant sex-related difference was found for prescribed postoperative anti-hypertension medications, such as beta blocker, angiotensin converting enzyme inhibitor/angiotensin receptor blocker, and calcium channel blocker (**Table 3**). Additionally, female patients in the type A AAD group primarily underwent ascending aorta replacement without the involvement of any other additional part (**Table 4**). Furthermore, the sex differences of the baseline characteristics were either negligible or small in the PSM cohort (**S1 Table**).

**Table 1:** Patient demographics and institution characteristics of the female and male patients who received AAD surgery before propensity score matching.

	Type A open				Type B stent			
Variable	Female (*n* = 1,286)	Male (*n* = 2,883)	*P*	STD	Female (*n* = 124)	Male (*n* = 549)	*P*	STD
Age (years)	65.1 ± 12.6	56.9 ± 13.1	<0.001	0.64	66.6 ± 11.8	63.2 ± 14.3	0.015	0.26
Monthly income, USD			<0.001				0.027	
0–596	461 (35.8)	886 (30.7)		0.11	36 (29.0)	202 (36.8)		-0.17
610–760	486 (37.8)	839 (29.1)		0.18	48 (38.7)	147 (26.8)		0.26
> 800	339 (26.4)	1,158 (40.2)		-0.30	40 (32.3)	200 (36.4)		-0.09
Urbanization level			<0.001				0.013	
Low	181 (14.1)	294 (10.2)		0.12	17 (13.7)	41 (7.5)		0.20
Moderate	395 (30.7)	834 (28.9)		0.04	40 (32.3)	150 (27.3)		0.11
High	359 (27.9)	953 (33.1)		-0.11	41 (33.1)	173 (31.5)		0.03
Very High	351 (27.3)	802 (27.8)		-0.01	26 (21.0)	185 (33.7)		-0.29
Surgical year			0.670				0.069	
Before 2007	362 (28.1)	833 (28.9)		-0.02	5 (4.0)	41 (7.5)		-0.15
2008–2010	425 (33.0)	920 (31.9)		0.02	18 (14.5)	112 (20.4)		-0.16
2011–2013	499 (38.8)	1,130 (39.2)		-0.01	101 (81.5)	396 (72.1)		0.22
Hospital level			0.771				0.338	
Medical center (teaching hospital)	989 (76.9)	2,229 (77.3)		-0.01	109 (87.9)	464 (84.5)		0.10
Regional / district hospital	297 (23.1)	654 (22.7)		0.01	15 (12.1)	85 (15.5)		-0.10
Previous cardiac surgery	41 (3.2)	143 (5.0)	0.010	-0.09	7 (5.6)	57 (10.4)	0.104	-0.18
Cumulative volume of aortic dissection surgery between 2004 and 2013			0.385				0.106	
1st quartile (1–132)	375 (29.2)	767 (26.6)		0.06	23 (18.5)	102 (18.6)		<0.01
2nd quartile (133–216)	305 (23.7)	722 (25.0)		-0.03	34 (27.4)	102 (18.6)		0.21
3rd quartile (220–345)	319 (24.8)	737 (25.6)		-0.02	19 (15.3)	78 (14.2)		0.03
4th quartile (355–687)	287 (22.3)	657 (22.8)		-0.01	48 (38.7)	267 (48.6)		-0.20
Follow-up (years)	2.8 ± 2.7	2.8 ± 2.7	0.772	-0.01	1.4 ± 1.6	1.8 ± 1.8	0.012	-0.26

AAD, acute aortic dissection; STD, standardized difference; USD, US dollar.

Value are given as number (%) or mean ± standard deviation.

**Table 2:** Comorbid conditions of the female and male patients who received AAD surgery before propensity score matching.

	Type A open				Type B stent			
Variable	Female (*n* = 1,286)	Male (*n* = 2,883)	*P*	STD	Female (*n* = 124)	Male (*n* = 549)	*P*	STD
Marfan syndrome	45 (3.5)	77 (2.7)	0.143	0.05	3 (2.4)	4 (0.7)	0.094	0.14
Hypertension	1,048 (81.5)	2,170 (75.3)	<0.001	0.15	101 (81.5)	480 (87.4)	0.080	-0.17
Diabetes mellitus	219 (17.0)	275 (9.5)	<0.001	0.22	27 (21.8)	78 (14.2)	0.036	0.20
Heart failure	88 (6.8)	162 (5.6)	0.124	0.05	9 (7.3)	37 (6.7)	0.836	0.02
Prior myocardial infarction	33 (2.6)	85 (2.9)	0.492	-0.02	7 (5.6)	30 (5.5)	0.936	0.01
Peripheral arterial disease	68 (5.3)	141 (4.9)	0.587	0.02	11 (8.9)	52 (9.5)	0.836	-0.02
Atrial fibrillation	109 (8.5)	166 (5.8)	0.001	0.11	4 (3.2)	16 (2.9)	0.854	0.02
Prior stroke	133 (10.3)	251 (8.7)	0.092	0.06	11 (8.9)	71 (12.9)	0.212	-0.13
Chronic kidney disease	206 (16.0)	585 (20.3)	0.001	-0.11	28 (22.6)	102 (18.6)	0.308	0.10
Liver cirrhosis	24 (1.9)	70 (2.4)	0.259	-0.04	4 (3.2)	15 (2.7)	0.764	0.03
Coagulopathy	23 (1.8)	47 (1.6)	0.713	0.01	1 (0.8)	10 (1.8)	0.421	-0.09
COPD	80 (6.2)	202 (7.0)	0.351	-0.03	10 (8.1)	63 (11.5)	0.270	-0.12
Depression	205 (15.9)	201 (7.0)	<0.001	0.28	20 (16.1)	53 (9.7)	0.036	0.19
Charlson’s Comorbidity Index score	2.4 ± 1.5	2.1 ± 1.5	<0.001	0.19	2.7 ± 2.0	2.4 ± 1.6	0.095	0.16

AAD, acute aortic dissection; STD, standardized difference; COPD, chronic obstructive pulmonary disease.

Value are given as number (%) or mean ± standard deviation.

**Table 3:** Medication use of the female and male patients who received AAD surgery before propensity score matching.

	Type A open				Type B stent			
Variable	Female (*n* = 1,286)	Male (*n* = 2,883)	*P*	STD	Female (*n* = 124)	Male (*n* = 549)	*P*	STD
Post OP anti-HTN medication								
ACEi/ ARB	429 (33.4)	957 (33.2)	0.917	<0.01	52 (41.9)	255 (46.4)	0.362	-0.09
Beta blocker	642 (49.9)	1,598 (55.4)	<0.001	-0.11	57 (46.0)	304 (55.4)	0.058	-0.19
CCB	474 (36.9)	1,135 (39.4)	0.124	-0.05	54 (43.5)	250 (45.5)	0.688	-0.04
Alpha-blocker	59 (4.6)	221 (7.7)	<0.001	-0.13	11 (8.9)	65 (11.8)	0.345	-0.10
Thiazide	46 (3.6)	75 (2.6)	0.083	0.06	9 (7.3)	15 (2.7)	0.014	0.21
Loop diuretics	327 (25.4)	643 (22.3)	0.027	0.07	18 (14.5)	56 (10.2)	0.165	0.13
Spironolactone (Potassium-sparing)	54 (4.2)	74 (2.6)	0.005	0.09	2 (1.6)	7 (1.3)	0.767	0.03
Vasodilator	184 (14.3)	388 (13.5)	0.461	0.02	20 (16.1)	111 (20.2)	0.299	-0.11
Nitrate	137 (10.7)	286 (9.9)	0.469	0.02	16 (12.9)	85 (15.5)	0.468	-0.07
Number of anti-HTN drugs	1.8 ± 1.6	1.9 ± 1.6	0.540	-0.02	2.0 ± 1.7	2.1 ± 1.5	0.300	-0.10
Post OP other medication								
Statin	63 (4.9)	116 (4.0)	0.198	0.04	8 (6.5)	59 (10.7)	0.149	-0.15
Antiplatelet	227 (17.7)	511 (17.7)	0.955	<0.01	31 (25.0)	167 (30.4)	0.232	-0.12
Anticoagulant	150 (11.7)	401 (13.9)	0.048	-0.07	1 (0.8)	15 (2.7)	0.204	-0.15
OHA	110 (8.6)	103 (3.6)	<0.001	0.21	16 (12.9)	33 (6.0)	0.008	0.24
Insulin	11 (0.9)	6 (0.2)	0.002	0.09	3 (2.4)	4 (0.7)	0.094	0.14

AAD, acute aortic dissection; STD, standardized difference; OP, operation; HTN, hypertension; ACEi, angiotensin converting enzyme inhibitor; ARB, angiotensin receptor blocker; CCB, calcium channel blocker; OHA, oral hypoglycemic agent.

Value are given as number (%) or mean ± standard deviation.

**Table 4:** Surgical characteristics of the female and male patients who received AAD surgery before propensity score matching.

	Type A open				Type B stent			
Variable	Female (*n* = 1,286)	Male (*n* = 2,883)	*P*	STD	Female (*n* = 124)	Male (*n* = 549)	*P*	STD
Type A dissection surgical detail (*n* = 4,169)								
Extension of aortic surgery								
Partial or total aortic arch replacement	354 (27.5)	949 (32.9)	0.001	-0.12	-	-		-
Aortic root replacement	106 (8.2)	312 (10.8)	0.010	-0.09	-	-		-
Elephant trunk	31 (2.4)	98 (3.4)	0.089	-0.06	-	-		-
Ascending aorta replacement only	817 (63.5)	1,593 (55.3)	<0.001	0.17	-	-		-
Additional surgery								
CABG	119 (9.3)	294 (10.2)	0.346	-0.03	1 (0.8)	11 (2.0)	0.363	-0.10
Valve replacement	119 (9.3)	258 (8.9)	0.752	0.01	1 (0.8)	6 (1.1)	0.776	-0.03

AAD, acute aortic dissection; STD, standardized difference; CABG, coronary artery bypass graft.

Value are given as number (%) or mean ± standard deviation.

### Sex difference in outcomes of type A open surgical repair

**Fig 2:**
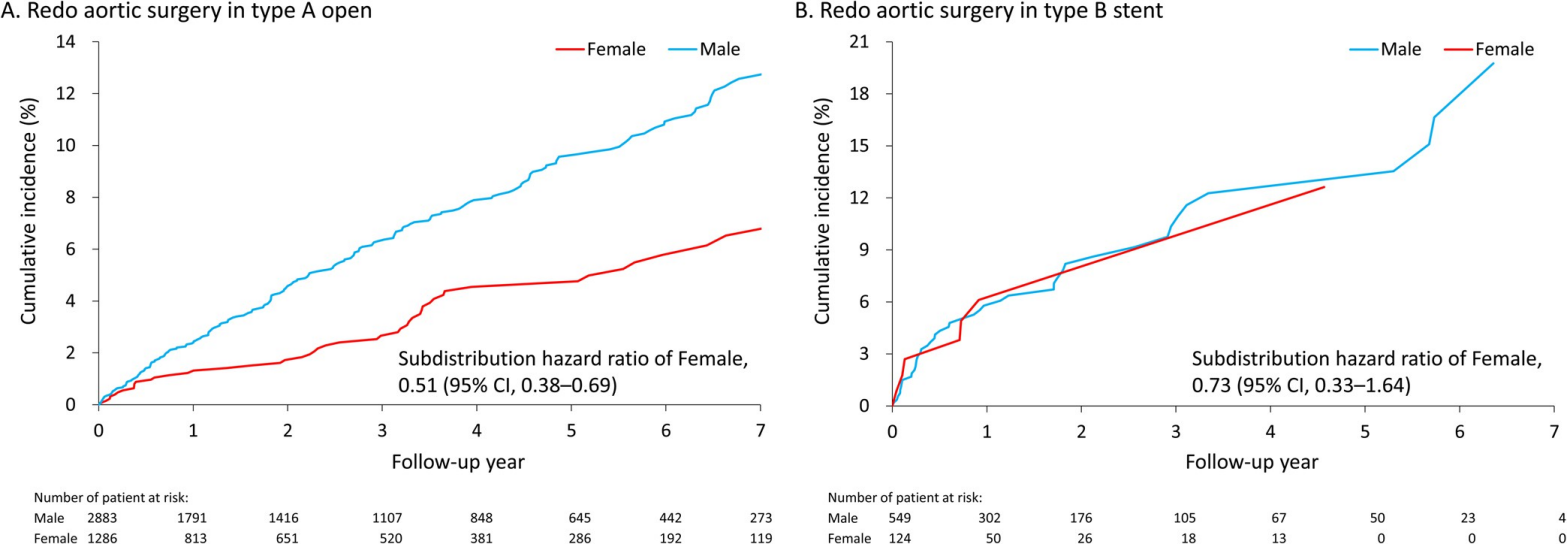
The cumulative incidence function of redo aortic surgery in the female and male patients who received type A open surgery (A) and type B stent surgery (B) by using the raw data for real-world practice. The subdistribution hazard ratio was not adjusted for any covariates.

**Fig 3:**
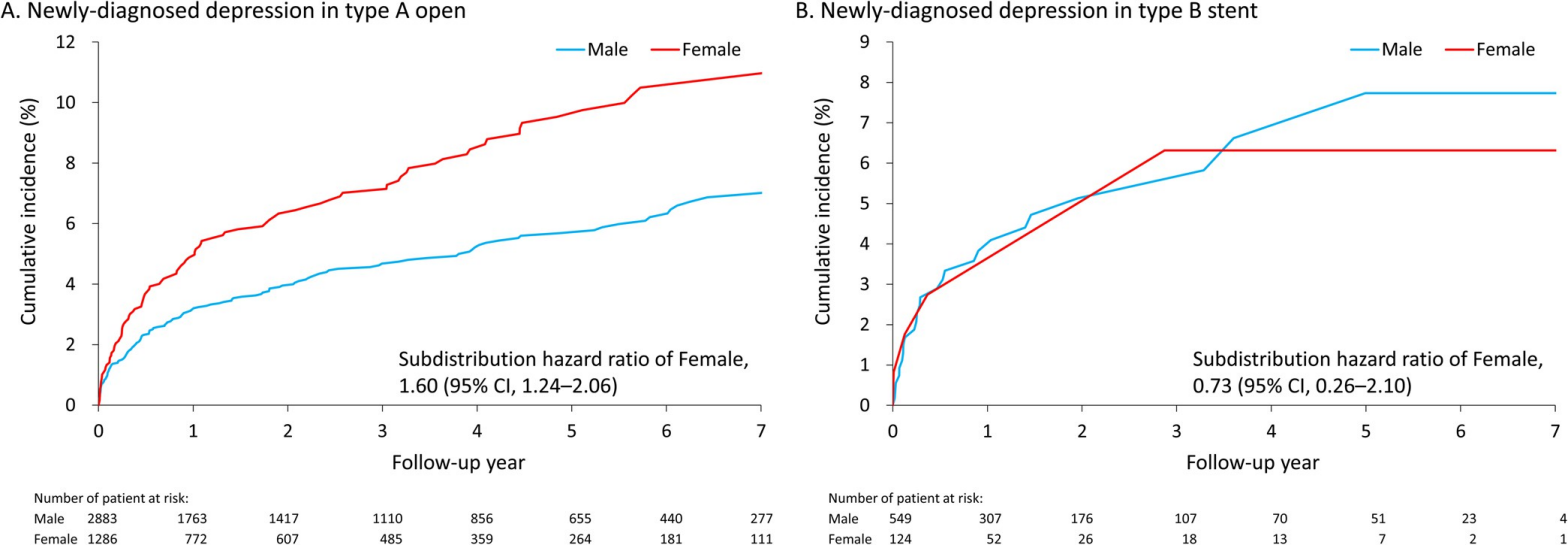
The cumulative incidence function of newly-diagnosed depression in the female and male patients who received type A open surgery (A) and type B stent surgery (B) by using the raw data for real-world practice. The subdistribution hazard ratio was not adjusted for any covariates.

**Table 5** shows the in-hospital outcomes of female and male patients following type A open surgical repair before PSM. No significant differences of all in-hospital outcomes were found between sexes. The mean follow-up duration was 2.8 years (standard deviation [SD]: 2.7 years). No significant difference in sex was found in the risk of all-cause mortality. However, the risk of redo aortic surgery was significantly greater in men than in women (7.8% vs. 4%; subdistribution hazard ratio [SHR] 0.51, 95% confidence interval [CI], 0.38–0.69) for type A open surgery (**Fig 2A**). Noticeably, the risk of newly-diagnosed depression in women was significantly greater than that in men (8% vs. 5.1%; SHR 1.6, 95% CI, 1.24–2.06) for type A open surgery (**Fig 3A**). The sensitivity analysis after PSM showed consistent results (**S2 Table**).

**Table 5:** In-hospital and long-term outcomes of the female versus male patients with type A open surgery before propensity score matching.

Outcome	Female (*n* = 1,286)	Male (*n* = 2,883)	OR/ *B* / HR or SHR of female (95% CI)
In-hospital outcome			
In-hospital mortality	251 (19.5)	590 (20.5)	0.94 (0.80–1.11)
New onset stroke	140 (10.9)	325 (11.2)	0.96 (0.78–1.19)
Massive blood transfusion†	429 (33.4)	1,008 (35.0)	0.93 (0.81–1.07)
Long-term outcome			
All-cause mortality	446 (34.7)	1,018 (35.3)	0.99 (0.88–1.10)
Redo aortic surgery	52 (4.0)	225 (7.8)	0.51 (0.38–0.69)*
Depression	103 (8.0)	147 (5.1)	1.60 (1.24–2.06)*

OR, odds ratio; *B*, regression coefficient; HR, hazard ratio; SHR, subdistribution hazard ratio; CI, confidence interval; PRBC, packed red blood cell.

† PRBC >10 Units.

* *P* < .05.

Value are given as number (%) or mean ± standard deviation.

### Sex difference in outcomes of type B stent surgery

**Table 6** shows the in-hospital outcomes of female and male patients following type B stent surgery before PSM. No significant differences in the risks of in-hospital mortality and massive blood transfusion were found between sexes. Noticeably, among the type B stenting treatment population, the postoperative incidence of stroke (female, 8.1% vs. male, 3.6%; odds ratio, 2.32; 95% CI, 1.06–5.09) in female patients was greater than that in male patients. The mean follow-up duration was 1.7 years (SD = 1.8 years). By contrast, no sex difference in the risks of long-term outcomes was observed (**Fig 2B and 3B**). The sensitivity analysis after PSM showed non-significant results (**S3 Table**). Otherwise, the in-hospital and long-term outcomes of patients with type B open repair were shown in **S4 Table**.

**Table 6:** In-hospital and long-term outcomes of the female versus male patients with type B stent surgery before propensity score matching.

Outcome	Female (*n* = 124)	Male (*n* = 549)	OR/ *B* / HR or SHR of female (95% CI)
In-hospital outcome			
In-hospital mortality	16 (12.9)	43 (7.8)	1.74 (0.95–3.21)
New onset stroke	10 (8.1)	20 (3.6)	2.32 (1.06–5.09)*
Massive blood transfusion†	17 (13.7)	45 (8.2)	1.78 (0.98–3.23)
Long-term outcome			
All-cause mortality	35 (28.2)	134 (24.4)	1.26 (0.87–1.82)
Redo aortic surgery	7 (5.6)	46 (8.4)	0.73 (0.33–1.64)
Depression	4 (3.2)	26 (4.7)	0.73 (0.26–2.10)

OR, odds ratio; *B*, regression coefficient; HR, hazard ratio; SHR, subdistribution hazard ratio; CI, confidence interval; PRBC, packed red blood cell.

† PRBC >10 Units.

* *P* < .05.

Value are given as number (%) or mean ± standard deviation.

## Discussion

This study analyzed the impact of sex difference on the perioperative and late outcomes of patients who received surgical management for AAD. We found that female patients were older when first diagnosed with AAD. No significant difference was found in the outcomes of either the in-hospital or all-cause mortality between sexes of both the type A open and the type B stent surgery populations. During the follow-up period, male patients were at higher risk of redo aortic surgery than female patients in the type A open surgery group. Remarkably, in the type A open surgery group, the risk of newly diagnosed depression in women was greater than that in men.

AAD is a potentially critical condition requiring emergent management. According to IRAD data, it is a male-dominant disease (66.9%) [1]. Because female patients with type A AAD have a propensity for poor postoperative outcomes, the reported surgical extent was relatively conservative [2, 17]. Female patients with type A AAD mostly underwent isolated ascending aorta replacement instead of complex surgical contents [18]. In our study, >80% of the female population had hypertension, which was considered as a major risk factor for AAD [19] (**Table 1**).

In type A AAD population, women had a higher postoperative mortality rate according to IRAD data [2, 20]. Nevertheless, several other recent cohort articles concluded no difference in either in-hospital mortality or 30-day mortality between female and male patients with type A AAD following emergent surgeries [3, 18, 21–23]. In the analysis of cumulative all-cause mortality, Smedberg et al. showed that female patients with type A AAD had no higher risk for long-term mortality after open surgery [20]. No significant difference in survival was indicated between the sexes for patients with type A AAD during the follow-up period in recent studies, analogous with our results [18, 23]. Similar to two Japan cohort studies, male patients who underwent type A AAD repair surgery had a significantly higher rate of aortic reoperation [23].

For patients with type B stenting treatment, no significant sex difference on in-hospital mortality was revealed in a cohort study, which was comparable to our result [24]. In our study, female patients undergoing type B stenting treatment had a higher incidence of new-onset stroke postoperatively, which was opposite to the cohort study using the National Inpatient Sample datasets showed that women had a lower incidence of acute stroke (females, 0.5% vs. males, 1.0; P = 0.01) [24]. The difference might arise from the baseline characteristics of study population. Compared with our enrolled population, the majority of patients with acute type B AAD population in the National Inpatients Sample datasets underwent open repair surgery instead of endovascular stenting treatment. No significant difference on long-term mortaliry after stenting management for patients with type B AAD was found between sexes [20].

Noticeably, in our study, a higher preoperative prevalence of depression (both type A and B AAD populations) and a significantly greater risk of newly diagnosed depression after index admission (type A AAD population) were found in women than in men. In the general population, the lifetime rate of depression for women was twice that for men [25, 26]. This discrepancy may be due to adaptive behavior, cognitive/social development, environmental influences (cultural stereotypes), and even brain/physiology differences (reproductive hormone fluctuations, hypothalamic–pituitary–adrenal axis regulation, and norepinephrine system adjustment) [27, 28]. The depression prevalence of female patients with AAD in our cohort was substantially higher than that in the general population. Women were potentially vulnerable to the stress response, which involved altered function of the hypothalamic–pituitary–adrenal axis and norepinephrine system, and further had implications for cardiovascular risk, especially coronary heart disease [6]. However, sex-related analyses of aortic diseases and depression was lacking. Future studies are needed to confirm the higher incidence of depression in women following open surgery for type A AAD.

### Limitations

The retrospective cohort nature limited this study. All the analyses were based on data obtained from the NHIRD, which does not record detailed information, such as vital signs, physical/laboratory examination data, or specific details of the imaging findings to obtain a follow-up diagnosis. In addition, aortic anatomy-specific data were unavailable, which are the leading factors affecting outcome. However, a panel review system of Taiwan’s Bureau of NHI is responsible for auditing payments of laboratory examination, medications, interventions, and surgeries. This review system could prevent abuse of medical resources and inappropriate indications for major interventions/surgeries, and further mitigate the associated bias.

The last year for NHIRD to release available data for research was 2013 because of the restriction of NHIRD. A lag time for years was required to clean up, correct, validate the data, and finally be released for study purposes. However, this study aimed to evaluate the sex-related difference following surgical treatment in specific AAD population. We supposed that the result would not be affected by the lack of more recent data from 2014 to 2019

Despite the limitations, the accuracy of NHIRD has been validated for analyses on patients with aortic dissection in a previous study [29]. Apart from our study, there had been several articles about the sex-related differences on the outcomes of AAD populations using big databases with varied results. Nevertheless, more further associated research are still demanded in the future.

## Conclusion

The morbidity and mortality rate for AAD patients following life-saving surgeries was improved gradually with time. In this study, there was no significant sex-related difference for the in-hospital mortality or accumulative all-cause mortality. However, higher incidence of redo aortic surgeries for male patients and new-diagnosed depression in female were found in type A AAD population following repair surgery. However, further associated research is demanded in the future to figure out the relationship between postoperative depression and female type A AAD patients.

## Supporting information

S1 Table Demographics, clinical and surgical characteristics of the female and male patients who received AAD surgery after propensity matching. (DOCX)

S2 Table In-hospital and long-term outcomes of the female versus male patients with type A open surgery after propensity score matching. (DOCX)

S3 Table In-hospital and long-term outcomes of the female versus male patients with type B stent surgery after propensity score matching. (DOCX)

S4 Table In-hospital and long-term outcomes of the type B open surgery versus type B stent surgery. (DOCX)
