# Supplementary material for: Sex-related differences on the risks of in-hospital and late outcomes after acute aortic dissection: A nationwide population-based cohort study
Source: PLoS One. 2022 Feb 10;17(2):e0263717. doi: 10.1371/journal.pone.0263717 (PMC8830652; doi:10.1371/journal.pone.0263717)
Supplement: S1 Table — (DOCX) [file pone.0263717.s001.docx]

**S1 Table.** Demographics, clinical and surgical characteristics of the female and male patients who received AAD surgery after propensity matching

|  | Type A open | | |  | Type B stent | | |
| --- | --- | --- | --- | --- | --- | --- | --- |
| Variable | Female  (*n* = 1,137) | Male  (*n* = 1,137) | STD |  | Female  (*n* = 113) | Male  (*n* = 113) | STD |
| Age (years) | 63.9 ± 12.5 | 63.9 ± 12.4 | -0.01 |  | 67.0 ± 12.1 | 68.8 ± 13.7 | -0.14 |
| Monthly income, USD |  |  |  |  |  |  |  |
| 0–596 | 393 (34.6) | 397 (34.9) | -0.01 |  | 35 (31.0) | 35 (31.0) | <0.01 |
| 610–760 | 421 (37.0) | 431 (37.9) | -0.02 |  | 40 (35.4) | 40 (35.4) | <0.01 |
| > 800 | 323 (28.4) | 309 (27.2) | 0.03 |  | 38 (33.6) | 38 (33.6) | <0.01 |
| Urbanization level |  |  |  |  |  |  |  |
| Low | 153 (13.5) | 162 (14.2) | -0.02 |  | 14 (12.4) | 12 (10.6) | 0.06 |
| Moderate | 350 (30.8) | 355 (31.2) | -0.01 |  | 36 (31.9) | 43 (38.1) | -0.13 |
| High | 324 (28.5) | 326 (28.7) | <0.01 |  | 37 (32.7) | 30 (26.5) | 0.14 |
| Very High | 310 (27.3) | 294 (25.9) | 0.03 |  | 26 (23.0) | 28 (24.8) | -0.04 |
| Surgical year |  |  |  |  |  |  |  |
| Before 2007 | 224 (19.7) | 229 (20.1) | -0.01 |  | 2 (1.8) | 3 (2.7) | -0.06 |
| 2008-2010 | 370 (32.5) | 377 (33.2) | -0.01 |  | 18 (15.9) | 16 (14.2) | 0.05 |
| 2011-2013 | 543 (47.8) | 531 (46.7) | 0.02 |  | 93 (82.3) | 94 (83.2) | -0.02 |
| Previous cardiac surgery | 41 (3.6) | 36 (3.2) | 0.02 |  | 6 (5.3) | 6 (5.3) | <0.01 |
| Comorbid conditions |  |  |  |  |  |  |  |
| Marfan syndrome | 36 (3.2) | 30 (2.6) | 0.03 |  | 2 (1.8) | 2 (1.8) | <0.01 |
| Hypertension | 917 (80.7) | 922 (81.1) | -0.01 |  | 95 (84.1) | 91 (80.5) | 0.09 |
| Diabetes mellitus | 172 (15.1) | 163 (14.3) | 0.02 |  | 25 (22.1) | 22 (19.5) | 0.07 |
| Heart failure | 75 (6.6) | 67 (5.9) | 0.03 |  | 9 (8.0) | 9 (8.0) | <0.01 |
| Prior myocardial infarction | 32 (2.8) | 30 (2.6) | 0.01 |  | 7 (6.2) | 9 (8.0) | -0.07 |
| Peripheral arterial disease | 55 (4.8) | 62 (5.5) | -0.03 |  | 10 (8.8) | 10 (8.8) | <0.01 |
| Atrial fibrillation | 89 (7.8) | 89 (7.8) | <0.01 |  | 4 (3.5) | 4 (3.5) | <0.01 |
| Prior stroke | 115 (10.1) | 120 (10.6) | -0.01 |  | 11 (9.7) | 10 (8.8) | 0.03 |
| Chronic kidney disease | 190 (16.7) | 194 (17.1) | -0.01 |  | 27 (23.9) | 31 (27.4) | -0.08 |
| Liver cirrhosis | 21 (1.8) | 22 (1.9) | -0.01 |  | 4 (3.5) | 3 (2.7) | 0.05 |
| Coagulopathy | 20 (1.8) | 15 (1.3) | 0.04 |  | 1 (0.9) | 0 (0.0) | 0.13 |
| COPD | 78 (6.9) | 81 (7.1) | -0.01 |  | 10 (8.8) | 15 (13.3) | -0.14 |
| Depression | 142 (12.5) | 133 (11.7) | 0.02 |  | 17 (15.0) | 17 (15.0) | <0.01 |
| Charlson’s Comorbidity Index score | 2.3 ± 1.5 | 2.3 ± 1.6 | <0.01 |  | 2.8 ± 2.0 | 2.9 ± 1.9 | -0.05 |
| Hospital level |  |  |  |  |  |  |  |
| Medical center (teaching hospital) | 874 (76.9) | 867 (76.3) | 0.01 |  | 100 (88.5) | 100 (88.5) | <0.01 |
| Regional / district hospital | 263 (23.1) | 270 (23.7) | -0.01 |  | 13 (11.5) | 13 (11.5) | <0.01 |
| Cumulative volume of aortic dissection surgery  between 2004 and 2013 |  |  |  |  |  |  |  |
| 1st quartile (1-132) | 329 (28.9) | 325 (28.6) | 0.01 |  | 19 (16.8) | 17 (15.0) | 0.05 |
| 2nd quartile (133-216) | 266 (23.4) | 273 (24.0) | -0.01 |  | 30 (26.5) | 25 (22.1) | 0.10 |
| 3rd quartile (220-345) | 282 (24.8) | 297 (26.1) | -0.03 |  | 18 (15.9) | 18 (15.9) | <0.01 |
| 4th quartile (355-687) | 260 (22.9) | 242 (21.3) | 0.04 |  | 46 (40.7) | 53 (46.9) | -0.13 |
| Post OP anti-HTN medication |  |  |  |  |  |  |  |
| ACEi/ ARB | 370 (32.5) | 372 (32.7) | <0.01 |  | 48 (42.5) | 43 (38.1) | 0.09 |
| Beta blocker | 576 (50.7) | 578 (50.8) | <0.01 |  | 55 (48.7) | 54 (47.8) | 0.02 |
| CCB | 426 (37.5) | 431 (37.9) | -0.01 |  | 49 (43.4) | 47 (41.6) | 0.04 |
| Alpha-blocker | 55 (4.8) | 55 (4.8) | <0.01 |  | 11 (9.7) | 9 (8.0) | 0.06 |
| Thiazide | 37 (3.3) | 39 (3.4) | -0.01 |  | 6 (5.3) | 5 (4.4) | 0.04 |
| Loop diuretics | 273 (24.0) | 284 (25.0) | -0.02 |  | 16 (14.2) | 15 (13.3) | 0.03 |
| Spironolactone (Potassium-sparing ) | 41 (3.6) | 42 (3.7) | <0.01 |  | 2 (1.8) | 2 (1.8) | <0.01 |
| Vasodilator | 155 (13.6) | 164 (14.4) | -0.02 |  | 20 (17.7) | 20 (17.7) | <0.01 |
| Nitrate | 117 (10.3) | 126 (11.1) | -0.03 |  | 16 (14.2) | 17 (15.0) | -0.03 |
| Number of anti-HTN drugs | 1.8 ± 1.6 | 1.9 ± 1.6 | -0.03 |  | 2.0 ± 1.8 | 1.9 ± 1.6 | 0.05 |
| Post OP other medication |  |  |  |  |  |  |  |
| Statin | 52 (4.6) | 53 (4.7) | <0.01 |  | 8 (7.1) | 8 (7.1) | <0.01 |
| Antiplatelet | 193 (17.0) | 189 (16.6) | 0.01 |  | 30 (26.5) | 33 (29.2) | -0.06 |
| Anticoagulant | 138 (12.1) | 134 (11.8) | 0.01 |  | 1 (0.9) | 0 (0.0) | 0.13 |
| OHA | 71 (6.2) | 76 (6.7) | -0.02 |  | 16 (14.2) | 15 (13.3) | 0.03 |
| Insulin | 4 (0.4) | 5 (0.4) | -0.01 |  | 2 (1.8) | 1 (0.9) | 0.08 |
| Type A dissection surgical detail (*n* = 4,169) |  |  |  |  |  |  |  |
| Extension of aortic surgery |  |  |  |  |  |  |  |
| Partial or total aortic arch replacement | 324 (28.5) | 324 (28.5) | <0.01 |  | - | - | - |
| Aortic root replacement | 90 (7.9) | 90 (7.9) | <0.01 |  | - | - | - |
| Elephant trunk | 28 (2.5) | 28 (2.5) | <0.01 |  | - | - | - |
| Ascending aorta replacement only | 713 (62.7) | 713 (62.7) | <0.01 |  | - | - | - |
| Additional surgery |  |  |  |  |  |  |  |
| CABG | 101 (8.9) | 102 (9.0) | <0.01 |  | 1 (0.9) | 0 (0.0) | 0.13 |
| Valve replacement | 103 (9.1) | 88 (7.7) | 0.05 |  | 1 (0.9) | 1 (0.9) | <0.01 |
| Follow-up (years) | 2.9 ± 2.7 | 2.8 ± 2.7 | 0.01 |  | 1.5 ± 1.7 | 1.6 ± 1.8 | -0.04 |

AAD, acute aortic dissection; STD, standardized difference; USD, US dollar; COPD, chronic obstructive pulmonary disease; OP, operation; HTN, hypertension; ACEi, angiotensin converting enzyme inhibitor; ARB, angiotensin receptor blocker; CCB, calcium channel blocker; OHA, oral hypoglycemic agent; CABG, coronary artery bypass graft;

Value are given as number (%) or mean ± standard deviation
