# Supplementary material for: Sex-related differences on the risks of in-hospital and late outcomes after acute aortic dissection: A nationwide population-based cohort study
Source: PLoS One. 2022 Feb 10;17(2):e0263717. doi: 10.1371/journal.pone.0263717 (PMC8830652; doi:10.1371/journal.pone.0263717)
Supplement: S2 Table — (DOCX) [file pone.0263717.s002.docx]

**S2 Table.** In-hospital and long-term outcomes of the female versus male patients with type A open surgery after propensity score matching

| Outcome | Female  (*n* = 1,137) | Male  (*n* = 1,137) | OR/ *B* / HR or SHR of female (95% CI) |
| --- | --- | --- | --- |
| In-hospital outcome |  |  |  |
| In-hospital mortality | 225 (19.8) | 222 (19.5) | 1.02 (0.83–1.25) |
| New onset stroke | 125 (11.0) | 111 (9.8) | 1.16 (0.87–1.53) |
| Massive blood transfusion† | 374 (32.9) | 370 (32.5) | 1.02 (0.85–1.22) |
| Long-term outcome |  |  |  |
| All-cause mortality | 394 (34.7) | 432 (38.0) | 0.91 (0.80–1.03) |
| Redo aortic surgery | 49 (4.3) | 75 (6.6) | 0.65 (0.46–0.93)* |
| Depression | 88 (7.7) | 64 (5.6) | 1.39 (1.01–1.91)* |

OR, odds ratio; *B*, regression coefficient; HR, hazard ratio; SHR, subdistribution hazard ratio; CI, confidence interval; PRBC, packed red blood cell;

† PRBC >10 Units;

* *P* < .05;

Value are given as number (%) or mean ± standard deviation.
