# Supplementary material for: Sex-related differences on the risks of in-hospital and late outcomes after acute aortic dissection: A nationwide population-based cohort study
Source: PLoS One. 2022 Feb 10;17(2):e0263717. doi: 10.1371/journal.pone.0263717 (PMC8830652; doi:10.1371/journal.pone.0263717)
Supplement: S3 Table — (DOCX) [file pone.0263717.s003.docx]

**S3 Table.** In-hospital and long-term outcomes of the female versus male patients with type B stent surgery after propensity score matching

| Outcome | Female  (*n* = 113) | Male  (*n* = 113) | OR/ *B* / HR or SHR of female (95% CI) |
| --- | --- | --- | --- |
| In-hospital outcome |  |  |  |
| In-hospital mortality | 15 (13.3) | 11 (9.7) | 1.42 (0.62–3.23) |
| New onset stroke | 9 (8.0) | 6 (5.3) | 1.55 (0.53–4.53) |
| Massive blood transfusion† | 17 (15.0) | 15 (13.3) | 1.15 (0.55–2.42) |
| Long-term outcome |  |  |  |
| All-cause mortality | 33 (29.2) | 37 (32.7) | 0.92 (0.60–1.39) |
| Redo aortic surgery | 7 (6.2) | 10 (8.8) | 0.72 (0.27–1.92) |
| Depression | 4 (3.5) | 7 (6.2) | 0.59 (0.17–2.02) |

OR, odds ratio; *B*, regression coefficient; HR, hazard ratio; SHR, subdistribution hazard ratio; CI, confidence interval; PRBC, packed red blood cell;

† PRBC >10 Units;

* *P* < .05;

Value are given as number (%) or mean ± standard deviation.
