# Supplementary material for: Sex-related differences on the risks of in-hospital and late outcomes after acute aortic dissection: A nationwide population-based cohort study
Source: PLoS One. 2022 Feb 10;17(2):e0263717. doi: 10.1371/journal.pone.0263717 (PMC8830652; doi:10.1371/journal.pone.0263717)
Supplement: S4 Table — (DOCX) [file pone.0263717.s004.docx]

**S4 Table.** In-hospital and long-term outcomes of the type B open surgery versus type B stent surgery

| Outcome | Type B stent  (*n* = 673) | Type B open  (*n* = 467) | OR/ *B* / HR or SHR of type B stent (95% CI) |
| --- | --- | --- | --- |
| In-hospital outcome |  |  |  |
| In-hospital mortality | 59 (8.8) | 80 (17.1) | 0.47 (0.32–0.67)* |
| New onset stroke | 30 (4.5) | 34 (7.3) | 0.59 (0.36–0.99)* |
| Massive blood transfusion† | 62 (9.2) | 152 (32.5) | 0.21 (0.15–0.29)* |
| Long-term outcome |  |  |  |
| All-cause mortality | 169 (25.1) | 178 (38.1) | 0.92 (0.74–1.14) |
| Redo aortic surgery | 53 (7.9) | 45 (9.6) | 1.35 (0.92–1.98) |
| Depression | 30 (4.5) | 34 (7.3) | 0.77 (0.47–1.26) |

OR, odds ratio; *B*, regression coefficient; HR, hazard ratio; SHR, subdistribution hazard ratio; CI, confidence interval; PRBC, packed red blood cell;

† PRBC >10 Units;

* *P* < .05;

Value are given as number (%) or mean ± standard deviation.
